# The intestinal digesta microbiota of tropical marine fish is largely uncultured and distinct from surrounding water microbiota

**DOI:** 10.1038/s41522-024-00484-x

**Published:** 2024-02-19

**Authors:** Melissa Soh, Ywee Chieh Tay, Co Sin Lee, Adrian Low, Laszlo Orban, Zeehan Jaafar, Henning Seedorf

**Affiliations:** 1 Temasek Life Sciences Laboratory, 1 Research Link, Singapore, 117604 Singapore; 2 Frontline Fish Genomics Research Group, Department of Applied Fish Biology, Institute of Aquaculture and Environmental Safety, Georgikon Campus, Hungarian University of Agriculture and Life Sciences, Keszthely, 8360 Hungary; 3 Department of Biological Sciences, National University of Singapore, Singapore, 117558 Singapore; 4 Present Address: Department of Medicine, Yong Loo Lin School of Medicine, National University of Singapore, MD6-Centre for Translational Medicine, 14 Medical Drive, Singapore, 117599 Singapore

**Keywords:** Microbiome, Microbial ecology

## Abstract

Studying the gut microbes of marine fishes is an important part of conservation as many fish species are increasingly threatened by extinction. The gut microbiota of only a small fraction of the more than 32,000 known fish species has been investigated. In this study we analysed the intestinal digesta microbiota composition of more than 50 different wild fish species from tropical waters. Our results show that the fish harbour intestinal digesta microbiota that are distinct from that of the surrounding water and that location, domestication status, and host intrinsic factors are strongly associated with the microbiota composition. Furthermore, we show that the vast majority (~97%) of the fish-associated microorganisms do not have any cultured representative. Considering the impact of the microbiota on host health and physiology, these findings underpin the call to also preserve the microbiota of host species, especially those that may be exposed to habitat destruction.

## Introduction

Collective scientific knowledge of host-associated microbial communities, also termed microbiota, has substantially increased over the last two decades^1–4^. Gut microbes form symbiotic relationships with the host and are intertwined with host survival, for example energy harvest^5,6^, immunity development^7,8^ and host defence against predation^9^, and/or infection^10^. Fish are the most diverse vertebrate organisms and tropical marine waters have some of the richest diversity of fish^11,12^. Yet not much is known about the gut microbiota, and specifically the intestinal digesta microbiota of many fish species, which are at risk of extinction from overfishing, global warming, and habitat destruction^13^.

Studies on fish-associated microbiota, typically in temperate regions, demonstrate the importance of extrinsic environment and intrinsic host factors to the gut microbiota^14,15^. Nonetheless, it would be imprudent to extrapolate this knowledge onto tropical fish. There are fundamental differences between temperate and tropical marine environments, including, but not limited to, environmental attributes^16^, host size differences^17^, and different species diversity^12^. These three factors have been separately found to influence gut microbiota^15,18,19^. In addition, diet has been found to affect gut microbiota in a range of fish species^20^. Given that the rates of herbivory in tropical marine fish are higher than that in temperate marine fish^21^, there is a fundamental difference in diet between fishes from different regions, which in turn indicates that there could be large differences between temperate and tropical marine fish gut microbiota. Consequently, patterns in host-associated microbes observed in temperate regions likely differ from tropical regions. This is especially so since most fishes are ectotherms, and water temperature is a crucial physical factor affecting fish growth and physiology^22,23^.

Southeast Asia is home to a remarkably high diversity of marine species^24–26^, making the region particularly interesting to study wild fish gut microbiota. Despite the small size, 618 shallow water marine fish species have been recorded in Singapore, of which few are introduced species^27^. In the Coral Triangle, it is estimated that there are 4350 marine fish species found^28^, while up to 3800 fish species have been reported for the adjacent South China Sea^29,30^. Severe anthropogenic disturbances in the region are likely to contribute to the ongoing habitat destruction and may lead to displacement or even extinction of species. However, despite degradation of Singapore’s natural coastal habitats, there is so far little/no decline in marine fish species richness^31^.

This study aims to understand the composition and factors affecting gut bacterial diversity in tropical marine fish. We used 16S rRNA gene amplicon sequencing to characterize the diversity of the digesta-associated bacterial gut microbiota, henceforth referred to as microbiota, that are associated with fish found in Singapore’s tropical marine waters. To do so, the relationship between water and fish host-associated microbes was explored. Next, we analysed the effect of environmental- and host-related factors on fish gut microbiota composition using both wild and farmed fishes.

Our results indicated that the microbiota of wild tropical fishes consists mostly of uncultured bacteria and they are distinct from the microbiota of the surrounding water, bringing to the forefront the untapped resource of diverse microbes with capabilities we can harness^32^. Furthermore, we tested the influence of multiple factors, specifically location, domestication status, host species, and trophic level, on microbiota composition, providing a general understanding of tropical marine fish gut microbiota, from which we can build upon to address problems faced by tropical aquaculturists^33,34^. Microbiota can also assist conservation efforts, since it has not only proven important to host health, but has also been found to confer adaptive potential to several wild species^35^. Altogether, these findings highlight the urgent need to protect the microbiome associated with tropical marine hosts.

## Results

**Fig. 1:**
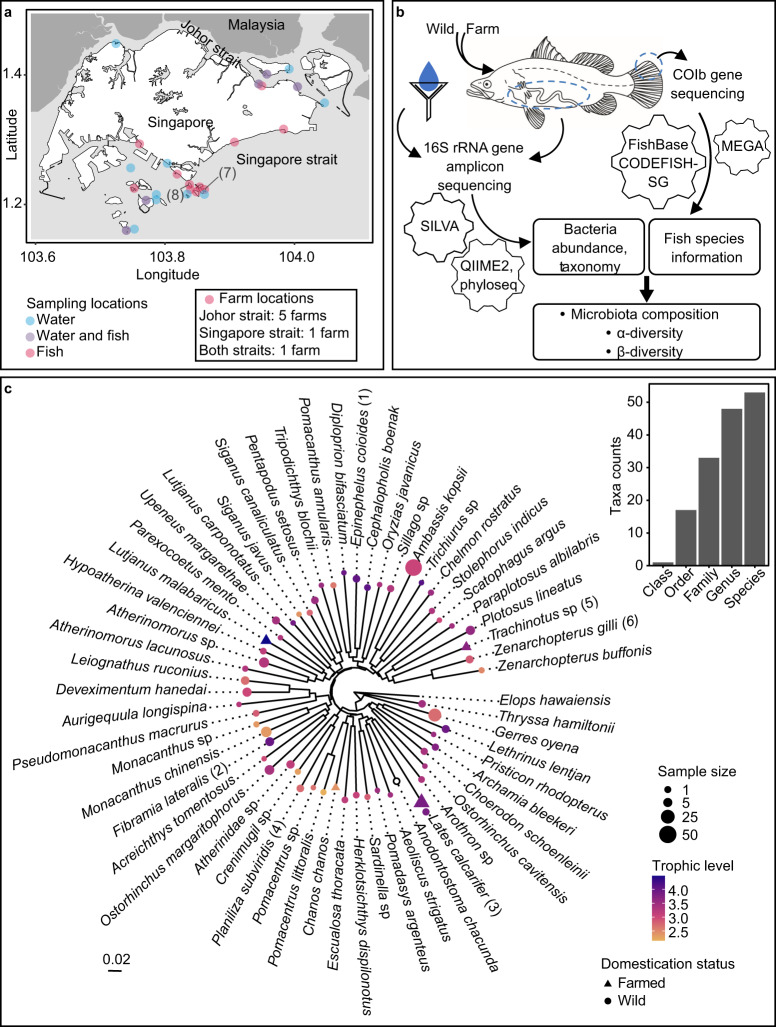
Overview of sampling location, methods, and resultant microbial and host diversity. **a** Map indicating sampling locations adapted from map by Wikimedia Commons contributors. Five farms are in Johor strait, one in Singapore strait, and one farm has locations in both Singapore and Johor straits. Specific locations of farms are not disclosed here. Numbers one to eight on panel A and C, in parenthesis, are reflected in Fig. 7. **b** Workflow illustrating main methods, programs, databases, analysis conducted. 16S rRNA gene amplicon sequencing was performed on water and fish gut contents. Sequencing results were processed using QIIME2, R package phyloseq, and compared against SILVA database. COI gene from host tissue was sequenced and processed using MEGA and compared with CODEFISH-SG database allowing identification of host species. Host ecology information were obtained from FishBase. Microbial composition, α- and β-diversity analysis were performed. Several icons used were derived from Microsoft PowerPoint. **c** Phylogenetic tree where size and shape of points indicate number of individuals sampled and domestication status respectively. Colour of points indicate host trophic level, with exception of white referring to juvenile fish. *Elops hawaiensis* was used as the outgroup. Bar plot on top right illustrates number of unique groups of fish, categorised by phylogeny.

A total of 282 wild bony fish specimens, representing 50 species in 31 families, were collected at 16 different locations within the territorial waters of Singapore (Fig. 1a, c, Supplementary tables 1, 2, and Methods for details). Fish were taxonomically classified using molecular barcodes (i.e. mitochondrial cytochrome oxidase subunit 1 (COI)) and morphological features. The 280 adult fish were designated trophic levels, ranging from 2.16 to 4.12, indicating that the fish sampled ranged from primary consumers to tertiary consumers i.e. herbivorous, omnivorous, and carnivorous. Trophic level of some fish species increase as they reach adulthood^36,37^. Thus, fractional trophic levels could not be confidently assigned to two juveniles and for microbiota analyses involving trophic level, only samples from adult fishes were used. A smaller number (*n* = 53, four species) of farmed fishes were also collected from seven local farms, as well as 100 water samples from 15 different locations for comparative analysis with the gut microbiota of wild fishes.

### Shared and unique microbes of the gut and water microbiota

**Fig. 2:**
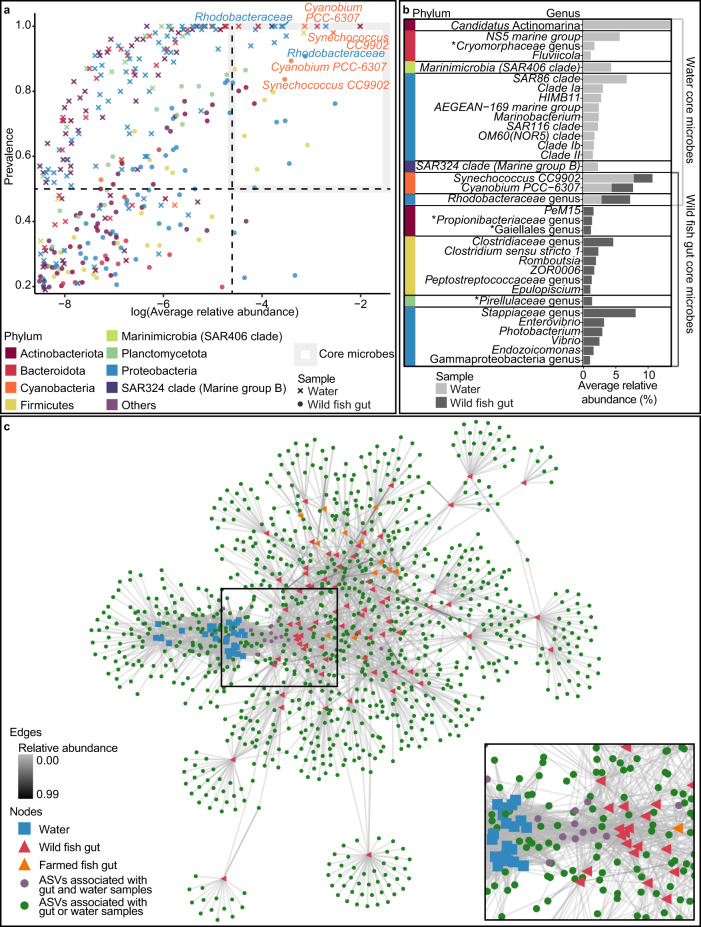
Comparative analysis of microbial composition and diversity associated with water and wild fish. **a** Relative abundance- prevalence plot representing genera from wild fish and water samples. Samples above the 85th percentile were illustrated in this plot. Grey lines demarcate threshold for core microbiota classification as per microbiome::core_members default criteria. Points are coloured by their respective phyla. Genera that appear in both wild and water samples after the cut-off point are labelled. Points are labelled with genus names, and if unavailable, family names. **b** Water and gut core microbiota relative abundance and phyla classification. **c** Network depicting relationship between samples and ASVs. Fish of same species and sampling location were grouped together. Water from same sampling location and season were grouped together. Portion of network enlarged and displayed on bottom right.

Fishes are constantly exposed to microorganisms from the surrounding waters. To investigate the relationship between microbiota associated with fish gut and water, we analysed the 16S rRNA gene amplicon sequencing data to determine their shared and unique microbiota (Fig. 2). Water samples were found to have 18 prevalent core genera (see Methods section for explanation of core genera detection), while wild fish gut had 19 core genera. Of these core genera, three were shared between water and wild fish gut (Fig. 2b). The shared genera are likely to be photosynthetic (*Synechococcus*, *Cyanobium*) or photoheterotropic (*Rhodobacteraceae* genus), which could indicate that they are transient. However, other highly abundant genera from the water, e.g. *Candidatus* Actinomarina and SAR86 clade were insufficiently abundant in gut samples to be considered core genera. Of the low-abundance ASVs, the ASV with the highest prevalence is a *Methyloceanibacter* at 83.3% prevalence (Supplementary Fig. 1).

To investigate the possible effects of location, wild fish gut and water samples were grouped by sampling strait. Regardless of sampling strait, Shannon index of water samples was significantly higher than that of gut samples from the same strait. Shannon index of gut samples from Johor strait was significantly higher than that from Singapore strait. This was also observed for water samples (Supplementary Fig. 2a). When comparing β-diversity between water and the five most sampled fish species in this dataset, water samples clustered away from fish samples, indicating a general distinction between the gut microbial communities (Supplementary Fig. 2b). The distinct differences in gut and water microbiota could also be observed at the amplicon sequence variant (ASV) level. Shared and unique microbiota at the ASV level were visualized using a network involving ASVs, wild fishes, farmed fishes, and water samples. The ASV network analysis indicates 15 ASVs shared between water and fish guts. However, the network highlights presence of many ASVs unique only to specific fish species and water samples (Fig. 2c). Neither ASVs nor genera specific to fish reached a prevalence higher than 60% (genus IMCC26207 (member of *Microtrichaceae*) had the highest prevalence at 57.80% and average abundance at 0.107%). While this may not exclude the presence of more prevalent core taxa below detectable levels, it indicates that these may only be present at very low relative abundance.

**Fig. 3:**
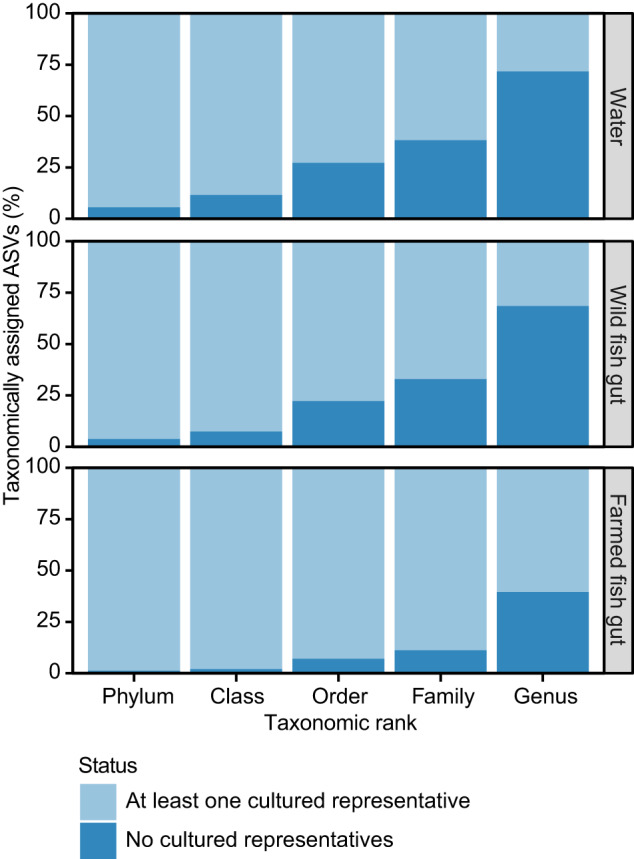
Contrasting cultured and uncultured taxa at different taxonomic ranks from three sample types. Taxa with at least one cultured representative are depicted against taxa with no cultured representatives. Water samples had a total of 8462 ASVs, wild fish gut samples had 19,723 ASVs, and farmed fish gut samples had 941 ASVs. Across taxonomic rank, water samples had the highest counts of taxa with no cultured representatives, followed by wild fish samples and then farmed fish samples.

Taxonomic assignment of ASVs to the genus level was only successful for a fraction of ASVs from water and wild fish samples (28.3% and 31.5% respectively). When there was no taxonomic assignment, we inferred that there are also no cultured representatives. Similar observations could be made for higher taxonomic ranks as no taxonomic assignment could be obtained for more than 30% of the ASVs at the family level (Fig. 3, see also Supplementary table 3 for details on cultured and uncultured taxa). This contrasts with the larger fraction of ASVs from farmed fish samples, which could be assigned to taxonomic ranks (genus 60.5%, family 88.8%).

### Trophic level is associated with fish gut microbiota and predicted microbiome composition

The presence of fish-specific microbiota prompted us to analyse factors that may potentially affect microbiota composition. Trophic level has been observed to influence the gut microbiota in a range of host species in other geographic regions^38^. To investigate potential interacting effects between host trophic level and the relationship between water and gut, the following analyses were performed.

**Fig. 4:**
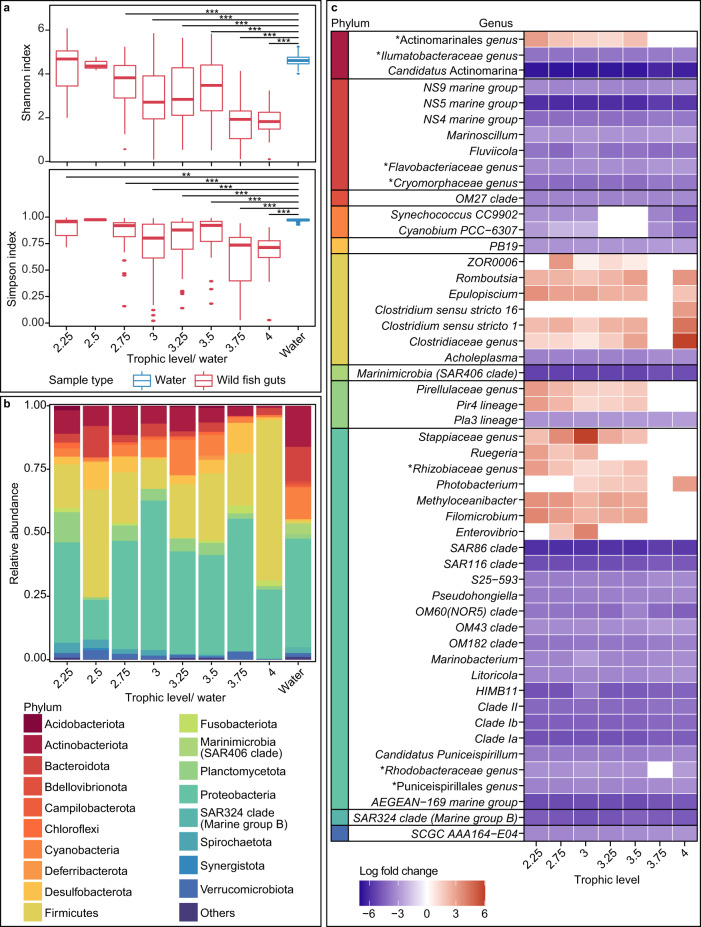
Comparing microbial diversity between water and wild fish gut samples across host trophic levels. Trophic level 2.25 samples (*n* = 22), 2.5 (*n* = 3), 2.75 (*n* = 58), 3 (*n* = 72), 3.25 (*n* = 53), 3.5 (*n* = 38), 3.75 (*n* = 8), 4 (*n* = 26), water samples (*n* = 100). **a** Boxplots indicating Shannon index and Simpson index of water and wild fish gut samples, with gut samples grouped by host trophic level. Wilcoxon rank sum test results for all comparisons against water samples indicated on plots. *P*-value ≤ 0.001: ***, ≤ 0.01: **, ≤ 0.05: *, and >0.05 is not significant and not displayed. Upper and lower hinges represent the 25th and 75th percentiles respectively. The upper and lower whisker connects the associated hinges to the highest and lowest value within 1.5 times of the interquartile range of the hinge respectively. Data points that were beyond the whiskers are considered outliers and plotted as points. **b** Stacked bar plot illustrating microbial phyla composition across host trophic level and water samples. **c** Heatmap depicting log fold changes of 51 genera across seven trophic groups. Genera arranged according to phyla, and legend is available in panel B. Water samples were used as reference group for ANCOM-BC calculations. Genera with log fold change greater than three or less than −3 in at least one trophic level, were included in this plot. * refers to unnamed bacteria.

First, the Shannon and Simpson index were calculated for each host trophic level group and for water samples, where host trophic level was grouped to nearest 0.25 (Fig. 4a). The results showed that the Shannon index was not significantly different between samples from low trophic level hosts and that of water but was significantly higher for water than all other host trophic level groups. The trend was similar when using Simpson index. Simpson index of samples from trophic level 2.5 hosts and water samples were not significantly different, while all other trophic groups had a significantly different Simpson index when compared to that of water samples. Additionally, increasing host trophic level was correlated with a decrease in the Shannon index (Pearson’s product-moment correlation: −0.366, *P*-value < 0.001), and decrease in low-abundance microbial ASVs (with less than 0.5% relative abundance in each sample; Pearson’s product-moment correlation: −0.352, *P*-value < 0.001). However, no obvious trends were found between host trophic level and water microbiota when considering other measures of microbial diversity (Supplementary Fig. 2c and Supplementary table 4 for power analyses).

The microbial composition was investigated at the phylum, and subsequently, genus level. We visualised microbiota composition at phylum level at different trophic levels (Fig. 4b) and performed ANCOM-BC analysis to identify genera that were differentially abundant between water and wild fish gut samples (filtering criteria are indicated in the Methods section). Of the 51 genera that met the filtering criteria, 16 genera showed higher log fold change in at least one trophic level group compared to water (Fig. 4c). These genera were from the phyla Actinobacteriota (1), Firmicutes (6), Planctomycetota (2), and Proteobacteria (7). All 15 core genera unique to water microbiota had negative log fold change in the wild fish gut of all host trophic level groups, while eight of the 16 core genera unique to wild fish hosts had positive log fold change values in at least two trophic level groups compared to water.

**Fig. 5:**
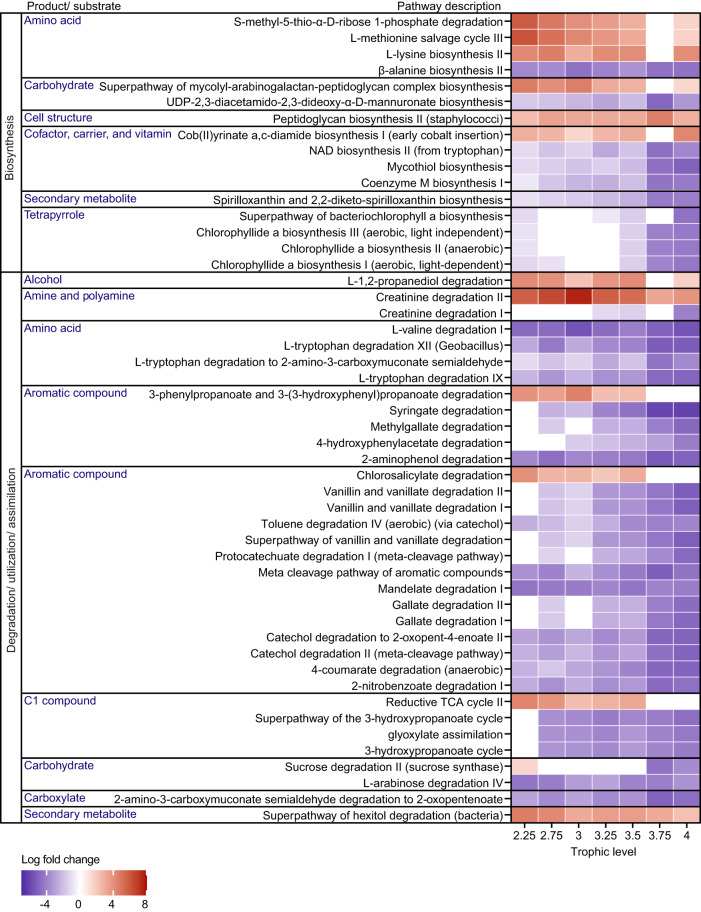
Functional analysis of microbiota across host trophic level. Log fold changes for 50 pathways across seven host trophic levels. Leftmost column indicates pathway categories based on Metacyc superclasses. Pathways were further classified by product or substrate involved. Water samples were used as reference group for ANCOM-BC calculations. Pathways with log fold change greater than four or less than −4 in at least one trophic level, were included in this plot.

Due to functional redundancy, changes in microbiota might not necessarily result in changes in functional traits of the microbiome as a whole. As such, changes in predicted pathway abundance across host trophic level was investigated. Three of the four pathways described as being involved in amino acid biosynthesis are predicted to occur more in fish gut microbiome than in water samples (Fig. 5). In general, there is a decrease in predicted pathway abundance as host trophic level increases, with the exception of two pathways (peptidoglycan biosynthesis II (staphylococci) and cob(II)yrinate a,c-diamide biosynthesis I (early cobalt insertion)) where higher abundance is predicted in the gut microbiome of high trophic level hosts. The reliability of the predictions is partially dependent on the availability of reference sequences for ASVs (see Supplementary Tables 5, 6 for specific ASVs contributions to pathway prediction). A calculation of mean NSTI for all ASVs indicated that these were within the range expected for less characterised environments^39^ (Supplementary Figure 3) (mean NSTI = 0.232). 41 of the 37581 ASVs from all fish and water samples imported to PICRUSt2 had a NSTI greater than two.

### Association of other host intrinsic parameters with fish gut microbiota composition

A subset of farmed fishes was included in the analysis to determine if domestication and other intrinsic factors contribute to gut microbiota composition. Farmed fishes face different environmental conditions compared to wild fishes. For instance, they may face lower predation stress and are frequently housed at higher densities than naturally occurring^40,41^. Simultaneously, host intrinsic parameters such as trophic level or phylogeny remain constant for each species, be it wild or captive. As such, comparing microbiota of farmed and wild fishes allows us to investigate the effect of host intrinsic parameters on gut microbiota. In general, each fish from the same sampling location experiences similar environmental factors. At the same time, each fish belonging to the same species would have similar host innate factors. Analysing sampling location and host species using PERMANOVA revealed that host innate factors had a bigger influence on the resultant microbiota. As such, further investigations into host intrinsic parameters were warranted.

Each host species experiences many host intrinsic parameters. Of these parameters, influence of host trophic level and evolutionary distance on microbiota were explored in previous studies of different population sizes and locations^38,42^. Here, after controlling for sampling location, trophic level was found to influence host microbiota more than evolutionary distance (sampling location R^2^: 19.21%, trophic level R^2^: 1.21%, evolutionary distance R^2^: 0.84%) (Table 1). On top of evolutionary distance and host trophic level, several other parameters were also investigated. After controlling for sampling location and trophic level, marginal effects of the following host intrinsic parameters were individually investigated: feeding path, type of parental care, pattern of parental care, standard length, weight, and aspect ratio. Of these parameters, both parental care parameters influenced microbiota to the greatest extend. PERMANOVA was also conducted where both parental care parameters were added sequentially to derive the impact of parental care strategy on microbial composition. Power analysis revealed that all statistical analysis involving grouped samples had power greater than 0.8 at 0.05 alpha significance level with the exception of the following: Bray-Curtis dissimilarity of wild and farmed samples grouped by type and pattern of parental care, host species, and host sampling location (Supplementary Table 4). As such, it has been noted that more samples of host exhibiting various types and patterns of parental care is required to confirm the trends observed. Furthermore, no host and location specific conclusions were made based solely on host species and sampling location.

**Table 1:** Comparing effect of variables on microbial diversity using PERMANOVA

Variables Tested	R^2^ (%)			
	1st Variable	2nd Variable	3rd Variable	4th Variable
Sampling Location * Host Species	7.51	24.97		
Sampling Location * Trophic Level* Evolutionary Distance	19.21	1.21	0.84	
Domestication Status	3.84			
Trophic Level	3.36			
Domestication Status * Trophic Level	2.37	1.88		
Sampling Location + Trophic Level + Feeding Path	23.49	1.36	1.16	
Sampling Location + Trophic Level + Type of Parental Care	23.49	1.36	1.86	
Sampling Location + Trophic Level + Pattern of Parental Care	23.49	1.36	2.62	
Sampling Location + Trophic Level + Type of Parental Care + Pattern of Parental Care	23.49	1.36	1.86	1.22
Sampling Location + Trophic Level + Standard Length	23.87	1.34	0.95	
Sampling Location + Trophic Level + Weight	27.52	1.21	0.61^	
Sampling Location + Trophic Level + Aspect Ratio	26.09	1.80	0.61	

When “*” is used between variables, marginal effect of each variable was derived. When “+” is used between variables, each variable was assessed sequentially from left to right. All *P*-values are 0.001 unless otherwise stated. ^*P*-value 0.002.

**Fig. 6:**
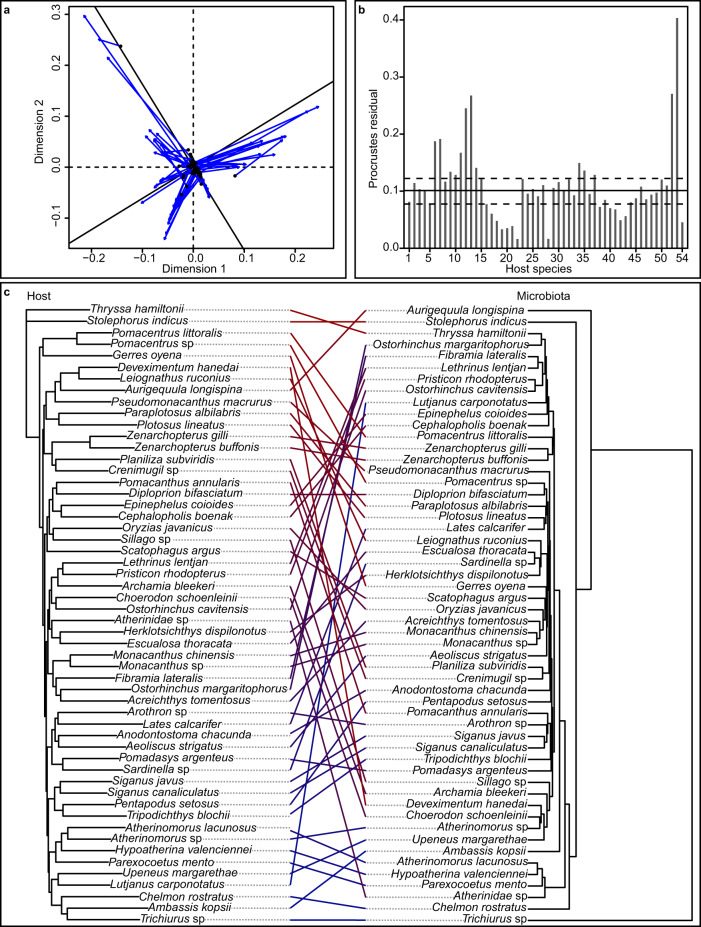
Phylosymbiosis between microbiota and wild fish hosts. **a** Blue vectors indicate the shift in points between the two configurations. Dotted line represents the axis of one configuration, while solid black line represents axis of second configuration. **b** An impulse diagram depicting residuals. Horizontal lines indicate 25% (bottom, dashed), 50% (middle, solid), and 75% (top, dashed) quantiles. Each bar represents residuals of one host species. Host species arranged based on order observed on host phylogenetic tree, starting from the top. **c** Host phylogenetic tree (left) and host microbiota hierachical clustering tree (right). Identical host species from both trees are linked together by lines coloured from red to blue, according to the order in host phylogenetic tree. Host tree plotted based on Kimura 2-parameter model. Microbiota hierachical clustering plotted based on weighted UniFrac distances.

The relationship between microbiota and host evolutionary distance was investigated using a phylosymbiosis analysis and visualised in Fig. 6c (with procrustes fit between host and microbiota configuration being depicted in Fig. 6a and procrustes residuals in Fig. 6b, respectively). Phylosymbiosis potentially provides insights on host-gut microbiota interactions that are affected by evolutionary processes^43^. Procrustes analyses using Weighted UniFrac distances of the microbiota indicated that sum of squares between host configuration and microbiota configuration was significant (Sum of squares: 0.9001, *p*-value < 0.05) (Fig. 6). When using either unweighted UniFrac or Bray-Curtis dissimilarities, there was no significant evidence observed for phylosymbiosis between microbiota and host species (both *P*-values > 0.05). Mantel test of weighted UniFrac distances between microbiota samples had a slight positive relationship with phylogenetic distance of fish host species (Mantel r: 0.2528, *P*-value < 0.05). Mantel test using Bray-Curtis dissimilarities for microbiota resulted in a significant but weaker relationship between host and microbiota distances (Mantel r: 0.1544, *P*-value < 0.05), while unweighted UniFrac distances between microbiota samples were not significantly related to distances between host species (Mantel r: 0.1265, *P*-value > 0.05).

Extrinsic and intrinsic parameters might not independently influence host gut microbiota. Since hosts experience a myriad of parameters at any given time, there could be an interplay between parameters in influencing the resultant microbial community. Parameters were first tested individually to obtain effect of each parameter, followed by tested together to obtain marginal effect of each parameter. The changes in R^2^ values when variables were tested together compared to individually indicate that there is interaction between parameters in influencing the microbiota. Interaction between trophic level and domestication status also affected α-diversity of gut samples. Farmed fish samples have a significantly lower α-diversity index than wild samples for both Shannon and Chao1 (*p*-value < 0.001) (Supplementary Fig. 4). Interacting effect of trophic level and domestication status on α-diversity was then analysed using linear mixed effects (LME) models. Interaction between trophic level and domestication was observed when comparing Shannon index of samples. Test estimate for interaction between trophic level and farmed fish is 0.1483 (standard error = 0.5368) while that for wild fish is −1.2028 (standard error = 0.2390).

**Fig. 7:**
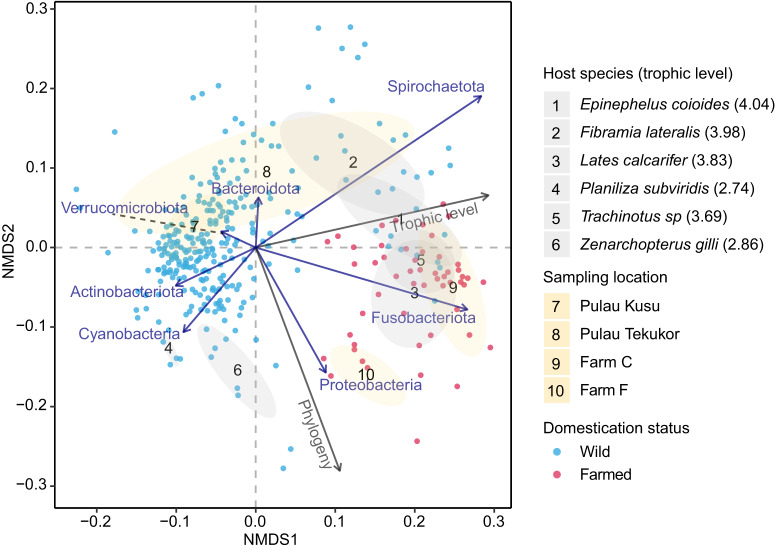
Clustering of samples and microbial gradients. NMDS calculated based on Bray- Curtis dissimilarity. Each number represents one centroid, and ellipses indicate standard deviation from centroid. Each grey ellipse represents one host species, and each yellow ellipse represents one sampling location. Six host species and four sampling locations were highlighted to maintain plot legibility, selected based on NMDS values. The ellipses represent farmed and wild samples, and trophic levels ranging from 2.74 to 4.04. Each ellipse represents at least five and at most forty individuals. Grey arrows indicate effect of trophic level and host phylogeny, and blue arrows indicate effect of seven phyla, selected based on NMDS values. (NMDS stress: 0.202, phylum-aggregated NMDS stress: 0.199).

A multidimensional perspective is required to investigate the relationship between samples with several parameters. To achieve that, NMDS comprising of wild and farmed fish samples was plotted (Fig. 7). NMDS reveals clustering of samples based on sampling location, host species, and domestication status and two gradients based on host trophic level and phylogeny. In addition, phyla gradients were observed across the NMDS. Six host species and four sampling locations were selected based on NMDS values and ellipses were drawn to illustrate clustering patterns. To visualise the effect of host trophic level on sample clustering, samples were coloured based on host trophic level (Supplementary Fig. 5a). For reference, Supplementary Fig. 5b includes all location ellipses, species ellipses, and phyla vectors.

## Discussion

Previous research into fish gut microbiota included freshwater and marine fishes from temperate regions^44–48^, freshwater fishes from tropical climates^49^, freshwater fishes living among varying degrees of anthropogenic activities^50^, and freshwater and marine captive conditions^51,52^. There are few previous research papers utilising culture independent techniques and involving wild tropical marine fishes^53–57^. These papers investigated factors affecting gut microbiota, changes in gut microbiota with time and space, and effects of aquatic compromise, using at most five species in one paper. This study, therefore, provides a baseline level of marine fish gut microbiota, which can be used for future studies or conservation efforts.

### Apparent host selection of gut microorganisms in the fish gut

This study supports the likelihood of host selection of gut microbiota as clusters of ASVs were unique to specific fish species from specific locations (Fig. 2c). Attempts to understand factors determining fish gut microbiota need to first consider host gut exposure to and colonisation by environmental microbes. Recent research analysed microbial communities within the Johor Strait, the water body north of Singapore island, a year after our water samples were collected, and found that microbial community structure exists in a relatively steady state with dominant bacterial taxa alternating between *Roseobacter* strain HIMB11, *Cyanobium* strain PCC6307, and *Saprospiraceae* related microbes^58^. Of these three taxa recovered from previous studies, strains HIMB11 and PCC6307 were core genera present in our water samples, indicating that these taxa are not only endemic to our water sampling locations but spread across Singapore coastal waters (Fig. 1a). However, 15 of the 18 water core genera were not considered in the core gut genera of the wild fishes studied, indicating host-selective processes that shape the microbiota composition. Other studies have observed similar effect of host species on gut microbial diversity but one study noted host habitat as a greater determinant^45,59^.

Wild fish gut associated microbiota are not only distinct from those that occur in the surrounding water, 68.1% of the ASVs have no apparent cultured genus-level representative (Fig. 3). A likely explanation for this may be that it has simply not been attempted yet to cultivate these microorganisms as their hosts remain poorly studied, while there may also be instances where it may be difficult or impossible to obtain cultures representatives as has been the case for *Candidatus* Epulopiscium. The high percentage of uncultured microorganisms hinders our ability to ascertain if wild tropical marine fish gut microbiota confers their hosts any adaptive potential. Nonetheless, it highlights the vast resource that is wild tropical marine fish gut microbiota. Conserving this microbial diversity would allow further investigation to uncover novel microbial enzymes and metabolites that could be of use^60^. Considering that this study includes 50 fish species of several thousands in the region alone, it is quite likely that the comprehensive characterization and cultivation of the tropical fish microbiome will be a major challenge. A review paper highlights the increasing need to either adapting the current nomenclatural framework or inventing a new framework to include uncultured taxa^61^. With growing use of high throughput sequencing, this solution might be inevitable.

Diet has been shown to shape gut microbiota composition in other animal groups and in farmed fish^62,63^. Gut microbes are able to digest food and extract nutrients previously inaccessible to the host^64^. Our research found that log fold change (LFC) of functional abundance indicate a general reduction in microbial activity in higher trophic level host guts. One possible reason could be that food of lower trophic level hosts require more processing to make nutrients available to the host^65^. Another possible reason could be the availability of niches in the host gut. Guts are often described as islands^66^. In addition, intestinal length of lower trophic level fish is generally longer than that of higher trophic level fish^67^. Species-area theory suggests that larger islands generally have higher species counts than smaller islands^68^. As such, the shorter gut length of high trophic level fish might influence the lower microbial diversity.

Another host intrinsic parameter, phylosymbiosis, has also been observed in insects, mammals, and fish, among other animal groups^43^. Compared to other host-associated microbiomes such as skin microbiome, gut microbiomes are uniquely suitable for investigations into phylosymbiosis between fish and their symbionts^49^. Previous publications investigated phylosymbiosis between fish and their gut microbiota with differing results. For instance, no patterns of phylosymbiosis were observed between twelve members of Sparidae and their gut microbiota^46^. Another study reported phylosymbiosis across all 24 freshwater fish species investigated^69^. Our study revealed that for tropical marine fish, when considering both microbial relative abundance and phylogenetic distances of gut microbiota, there is a slight positive phylosymbiotic relationship. This observation, despite the use of intestinal digesta microbiota instead of other host associated microbiota, might explain why previous studies involving fewer samples and/or fewer host taxa had differing conclusions^70^. As future studies increase the number of host species investigated, more information on the drivers of phylosymbiosis can be revealed.

Previous research in other species indicates that environment^45^ and domestication status^71^ influence host gut microbial community. In addition, host trophic level is regarded as a driver of microbial composition in fish^72^. We observe a similar trend as Shannon and Simpson index, microbial diversity, and functional prediction are associated with changes in host trophic level. Regarding α-diversity, Shannon index values decrease with increasing host trophic level for wild but not farmed fishes. This trend was not observed when measuring α-diversity using Chao1 index implying that interactions between host trophic level and domestication status influences microbial composition evenness. Interaction between extrinsic and intrinsic parameters were also observed when investigating β-diversity using Bray-Curtis dissimilarity.

The NMDS-based visualization revealed that fish samples were first clustered based on domestication status, and thereafter on fish species. Based on PERMANOVA tests, effect of host trophic level and domestication on β-diversity changes depending on whether factors were analysed together or singly, indicating the interplay between factors in microbiota assembly. It should be noted that more species of farmed fish from varying trophic levels are needed to confirm the presence of this interaction. In contrast, predicted functional analysis revealed that the highest host trophic level analysed here corresponded to higher cob(II)yrinate a,c-diamide (Vitamin B_12_) biosynthesis. Low trophic level fish consume algae which contain Vitamin B_12_ and thus might rely less on gut bacterial Vitamin B_12_ biosynthesis^73^. In addition, a previous review indicates that there could be more cobalamin in microalgae than in fish meal^74^. This might explain the predicted lower cob(II)yrinate a,c-diamide biosynthesis functional abundance in the gut microbiota of lower trophic level host compared to higher trophic level host. While these predictions may allow a glimpse into the metagenomic potential, it needs to be noted that these predicted functional analyses have known limitations. For example, the confidence of predictions depends on the availability of close reference sequences and predictions for taxa that are not covered by amplicon PCR are completely omitted. Future studies should therefore be complemented with metabolomics and/ or metatranscriptomics, which may be more feasible for some species than others due to availability, size/ amount of sample from the specimens, etc.

Lastly, previous publications found that fish first obtain microbes by transfer from parental microbiota^75,76^, by consumption of microbiota residing on egg surface upon hatching^77,78^, or by food sources^79,80^. Current findings indicate that method of parental care, host trophic level, and sampling location influences gut microbial diversity, and are thus in line with previous findings. More samples from different parental care types are required to obtain more comprehensive insights.

Differences between fish gut associated and free-living marine microbial communities were observed when comparing α-, β-diversity, and microbial composition of wild tropical marine fish. This indicates that tropical marine fish gut microbiota assemblage is unlikely to be stochastic and that host selection and enrichment drive the resultant microbial composition. Comparison of the effect of sampling location extrinsic parameters and host species intrinsic parameters on β-diversity wild tropical marine fish microbiota revealed that the latter is of greater influence. Investigations into specific host intrinsic parameters identified several main drivers of β-diversity, namely, host trophic level and potentially method of parental care. Host trophic level also influenced microbial composition and α-diversity. In addition, evidence suggests that extrinsic and intrinsic parameters interact in determining the microbial composition. Factors explored in this paper do not explain gut microbial diversity within fish species. Further research into gut microbial diversity of one species of tropical marine fish would allow us to control for multiple extrinsic and intrinsic parameters, thus elucidating potential host intrinsic parameters more specific than host species.

## Methods

### Fish sample collection

All wild fish sampling was subject to approval by the Institutional Animal Care and Use Committee (IACUC) of the National University of Singapore. The full proposal for MSRDP project 18 was considered by the IACUC ethics committee, which approved the Biodiversity Protocol under protocol number B17-1123. Wild fishes were collected from sixteen sites around Singapore under Permit No:NP/RP18-051b (*n* = 282) (Fig. 1a). Farmed fish samples were collected from seven locations (*n* = 53). Fishes were buried in ice immediately after collection and transported to a −80 °C freezer where they were stored until dissection. Sterile dissecting implements were used to extract guts from host fishes. Gut contents from the entire gut (stomach to rectum) were squeezed into sample tubes to reduce amount of host DNA in resultant DNA extracted. For fishes weighing more than 100 g, contents from hindgut were homogenised and sampled.

### Determination of host species identity

For every gut sampled, host genomic DNA from fin clippings was extracted using a bead-beating phenol-chloroform extraction method^81^. COI gene amplicons were then generated. Each PCR reaction contained 12.7 µl 1× GoTaq MasterMix (Promega), final concentration of 0.43 µM forward primer Ill_B_F (5′CCNGAYATRGCNTTYCCNCG3′)^82^, final concentration of 0.43 µM reverse primer FishR12 (5′ACTTCWGGGTGRCCRAAGAATCA3′), DNA template, and molecular grade water. FishR12 was designed based on FishR1 and FishR2^83^. In negative controls, DNA template was replaced by sterile molecular grade water. Bio-Rad thermal cyclers were used to achieve the following PCR conditions: 95 °C for 5 min, 35 cycles of 94 °C for 30 s, 56 °C for 60 s, 72 °C for 40 s, and finally, elongation at 72 °C for 5 min. PCR products were loaded into an agarose gel, and amplicons were pooled based on resultant band intensity. The pooled amplicons were purified using 0.9 volume AMPure™ XP (Beckman Coulter Genomics, Danvers, MA, USA) and then sequenced on Illumina NovaSeq PE250 platform. Pear (version 0.9.6) was used to assemble forward and reverse reads with options set at “-q 25 -m 480 -n 475 -v 20”. Demultiplexing was done using Python script NGSbarcoder_mult_1.1.py^84^. Local BLAST analysis of the demultiplexed samples was done against a curated in-house reference database. In brief, the database was generated as following: Fish and their life stages were identified by local ichthyofauna experts, which used morphological features of the fish for identification. Verified fish species were crossmatched with cytochrome c oxidase subunit 1 sequencing data that were generated for this project. Fishes that could not be identified to species were identified to genus or family level (nine genera, one family). Fish were also identified using morphological features.

### Determining host related parameters of each fish species

Trophic level, two reproductive guilds, feeding path, host aspect ratio, evolutionary distance, host standard length, and host weight were used in data analyses. Of these parameters, the last two details were measured from individual hosts while the remaining six were derived as described in the following paragraphs.

R package rfishbase version 3.1.6 was paramount in deriving the first five host related parameters^85,86^. Host fractional trophic level was used to quantify host diet for all adult species. *Anodontostoma chacunda* specimens collected were juveniles and thus trophic level were not assigned^87^. Trophic level was obtained using the rfishbase::estimate under “Troph”. For samples that were identified only to genus or family level, trophic levels of members in the same taxa were averaged. Each fish species reproductive guild information was derived using rfishbase::reproduction, under “RepGuild1” and “RepGuild2”. RepGuild1 describes type of parental care (non-guarders, guarders, and bearers). RepGuild2 describes pattern of parental care (egg scatterers, nesters, external brooders, and internal live bearers). Feeding path (pelagic or benthic) was derived from rfishbase::estimate under “FeedingPath”. When reproductive guild and feeding path data were unavailable, data from a higher taxonomy level was used, up to family level, after which an ichthyologist was consulted. Aspect ratio of caudal fin (height of fin^2^/surface area of fin) was derived rfishbase::morphometrics under “AspectRatio”. Where multiple aspect ratios were recorded per fish species, the mean was used (see Supplementary Table 1 for fish metadata).

Kimura 2-parameter (K2P) distances were used as a measure of host genetic distance^88^. COI amplicon sequences were aligned using ClustalW in MEGA X programme (Molecular Evolutionary Genetics Analysis) version 10.2.4^89,90^. MEGA X was then used to calculate K2P distances between each host species and *Elops hawaiensis* outgroup (intraspecies K2P pairwise distance mean = 0.956% ± 2.933 (standard deviation) within acceptable range^91–93^). A neighbour-joining phylogenetic tree was also constructed following K2P model^94^ (Fig. 1c).

### Fish gut sample processing for gut microbiota analysis

Genomic DNA was extracted from the gut samples using a bead-beating phenol-chloroform extraction method and 0.1 mm diameter zirconia beads^81^. Quant-it Picogreen (Thermo Fisher Scientific, OR, USA) was then used to quantify gDNA obtained, allowing equal amounts of DNA to be added to each PCR reaction. 16S rRNA gene V4 region was amplified using single index PCR with 515 F and 806 R primers as described in the 16S Illumina Amplicon Protocol from the Earth Microbiome Project^95–97^. Each sample was amplified in triplicates, and each single index primer pair was tested with a template-free negative control. In addition, mock microbial community ABRF-MGRG 6 Strain Even Mix Genomic Material (ATCC MSA-3000) was also amplified using the same protocol. Each PCR reaction contained 5 μl 5x New England BioLabs Q5 buffer, final concentration of 80 μM of Promega U1515 dNTP mix per nucleotide type, 0.4U New England BioLabs Q5® High-Fidelity DNA polymerase, forward and reverse primers at final concentration of 0.2 µM each, 30 ng gDNA template, topped up to 25 µl using sterile molecular grade water. Bio-Rad thermal cyclers were set at the following conditions: 94 °C for 3 min, 34 cycles of 94 °C for 45 s, 50 °C for 60 s, 72 °C for 1:30 min, and finally, elongation at 72 °C for 10 min. Resultant PCR product triplicates were pooled together and ran on 2% agarose gel. The pooled triplicates were subsequently pooled together according to DNA band intensity visualised from the agarose gel. Pooled samples were purified using AMPure™ XP (Beckman Coulter Genomics, Danvers, MA, USA). DNA quality and quantity was measured using Nanodrop and Qubit. Pooled PCR products were sequenced at Genome Institute of Singapore on the Illumina Miseq platform using paired-end sequencing chemistry. Sequencing was done using either the 251 bp or 151 bp sequencing platform.

### Water sample collection

Water sampling was conducted during March and June 2019, timed to coincide with Singapore inter-monsoon and monsoon seasons respectively. During each season, a horizontal Van Dorn water sampler was used to sample water from 15 locations around Singapore (Fig. 1a). These locations covered four different habitat types: mangrove, seagrass, coral reef, and open channel. Locations were accessed via boardwalks or small vessels, where at each location, 2–3 L of water were collected from each of three sites ~50 m apart (see Supplementary Table 2 for water metadata). Water samples were immediately stored on ice until vacuum-filtered at 60kPa using 0.22 µm nylon membranes (Nalgene, NLG#DS0215 − 4020). Subsequently, filter membranes were stored at −80 °C until DNA extraction.

### Water samples eDNA extraction and metabarcoding

DNA was extracted from the filter membrane using a phenol-chloroform method as described above. The following modifications were necessary to accommodate for the difference in sample type. Prior to bead beating, each filter paper was placed in an 8 ml tube that contains autoclave sterilized 0.4 g zirconium beads and 2 mg proteinase K in 1 ml CTAB. These tubes were incubated at 55 °C for 2.5 h, shaking at 250 rpm in an Innova 4000 Incubator Shaker to wash filtration residue off filter membrane. Instead of using QIAquick 96 PCR Purification Kits (Qiagen), extractions were rested at 4 °C overnight, then centrifuged at 19,500 r.c.f. for 20 min at 4 °C. Resultant DNA pellets were washed with 80% ethanol twice, airdried ~4 h, then dissolved in. molecular grade water. 16S rRNA gene V4 region was amplified and processed in a similar manner as described for gut samples, with 8 µl 10–120× diluted DNA.

### Amplicon sequence processing and microbiota analysis

16S rRNA gene amplicon sequencing results from both water and fish gut samples were processed in the same manner. Demultiplexed fastq files were processed using QIIME 2 2021.4^98^. Forward reads were imported into QIIME 2 using “qiime tools import” command, with options “--type ‘SampleData[SequencesWithQuality]’” and “--input-format SingleEndFastqManifestPhred33”. Reads were denoised, dereplicated, chimera filtered and trimmed using command “qiime dada2 denoise-single”, with options “--p-trim-left 12” and “--p-trunc-len 150”^99^. Each sequencing run was processed individually. Resultant feature tables were merged using command “qiime feature-table merge” and option “--p-overlap-method sum”. Representative sequences were merged using “qiime feature-table merge-seqs”. ASV taxonomic identity was assigned using command “qiime feature-classifier classify-sklearn” and SILVA 138 SSU 99% identity 16S 515 F/806 R region database^100^. “qiime feature-table filter-seqs” was then used to filter the representative sequences according to the feature-table output from the previous command. Resultant representative sequences were aligned using command “qiime phylogeny align-to-tree-mafft-fasttree”. Alpha rarefaction curves were generated using “qiime diversity alpha-rarefaction” at “--p-max-depth 20,000”. The output was visualised at https://view.qiime2.org/^101^. Based on the plateau observed on alpha rarefaction curves, rarefaction depth of 6,472 was chosen and diversity metrics were generated using commands “qiime diversity core-metrics-phylogenetic”.

### Overview of statistical methods

Statistical tests, modelling, and visualisation were conducted in R^102^. Output from QIIME2 was imported into R and processed using phyloseq package, while visualisations were made using ggplot2^103,104^. Power analyses were performed using QIIME2 Evident plugin and QIIME 2 version 2023.9 for each of the statistical tests involving grouping samples. “qiime evident univariate-power-analysis” was used for power analysis of statistical tests involving α- diversity, while “qiime evident multivariate-power-analysis” was used for that of β- diversity^98,105^. When trophic level was unavailable (*n* = 2), affected gut samples were excluded from trophic level related analysis. When necessary, grouping of samples by trophic level were done by rounding to nearest 0.25. All logarithm calculations were done using natural logarithm. All Wilcoxon rank sum tests conducted utilised Benjamini-Hochberg *P*-value adjustment method.

### Microbial composition analysis

A variety of R packages were used in data analysis. Core microbes for each sample type were identified using core_members function in R microbiome package, and default thresholds (detection threshold: 1%, prevalence threshold: 50%)^106^ were used as recently suggested by Neu et al.^107^. In stacked barplot depicting phyla relative abundance, phyla with less than 0.5% relative abundance each in all groups of samples have been labelled as “Others” using R package genefilter^108^.

To generate a network, gut samples were grouped by host species and sampling location, while water samples grouped by sampling location and sampling season. After grouping, ASV abundances were transformed to relative abundance per grouped sample. After network generation, network was filtered to only include edges corresponding to ASV relative abundance greater than 0.5%. Network was then displayed using Prefuse Force Directed OpenCL Layout. Cytoscape 3.9.1 and R package Rcy3 were used in network generation^109,110^.

PICRUSt2 (v2.3.0-b) was used to predict MetaCyc metabolic pathways of water and wild fish gut microbiota^39,111^. The default pipeline “picrust2_pipeline.py” was used, following which “add_descriptions.py” was ran, resulting in output files that indicates pathway abundance and description. Nearest Sequenced Taxon Index (NSTI) for each ASV, mean NSTI for each host species or water sample, and mean NSTI for all ASVs were calculated and visualised (Supplementary Fig. 3). ASVs with NSTI greater than two were removed from further analysis as per default in PICRUSt2.

Analysis of Compositions of Microbiomes with Bias Correction (ANCOM-BC) was performed using water and wild fish microbiota aggregated to the genus level. The ancombc function from ANCOMBC package^112^ was used with all default settings, except the false discovery rate adjusted p-value threshold set at “alpha = 0.001”, and since a phyloseq object was used, assay_name was set as NULL. Formula was set to be trophic level. Water samples were used as the reference group. Samples from host trophic level 2.5 group were left out of this analysis due to ANCOM-BC sample size requirement. To analyse and visualise genera with greater changes in abundance, taxa were filtered based on LFC, where only genera with maximum LFC greater than three or minimum LFC less than −3 in at least one trophic level group were included in the resultant figure (Fig. 4c). ANCOM-BC was also performed to identify differentially expressed metabolic pathways across host trophic levels, using the same ancombc arguments as described above (Fig. 5). The abundance of pathways identified by PICRUSt2 necessitated a stricter LFC filtering threshold when selecting pathways to visualise. As such only pathways with maximum LFC of greater than four or minimum LFC less than −4 in at least one trophic level group were included in the figure. Pathways were categorised based on Metacyc.

For phylosymbiotic analysis, microbiota of each gut sample was grouped together based on host species, then transformed to relative abundance. Phyloseq package distance function was used to calculate distance between resultant gut microbiota samples, based on weighted UniFrac, UniFrac, and Bray-Curtis^103^. Host phylogenetic tree was calculated as described above, except that only wild samples and no outgroup was incorporated this time. Distances were then imported into R. Configurations for both host and microbiota were calculated based on dissimilarities using cmdscale function^102^. Configurations were compared using procrustes function from ecodist package, and plotted using the plot function^113^ (Fig. 6a, b). Procrustes sum of squares were calculated using protest function and 999 permutations. Mantel test was conducted using mantel function. Microbial hierarchical clustering was determined using hclust function on weighted UniFrac distances^102^ and visualised against host phylogenetic tree (Fig. 6c).

### Analysis of α- and β-diversity

Relationship between extrinsic factors, intrinsic host factors, and two α-diversity indices (Shannon index, Chao1 index) of the samples was investigated by modelling using R package lme4^114^. Information theory was used in model selection. All models were checked to ensure that they meet the LME assumptions of homoscedasticity and normal error distribution. Models were fitted based on dataset containing both farmed and wild fish samples. R visreg package was used to visualise the models^115^.

In NMDS plots, points, centroids, ellipses, and continuous variables (host trophic level and host evolutionary distance) were plotted in ASV resolution using vegan and BiodiversityR packages^116,117^. To calculate microbe phylum level vectors, taxonomic data were aggregated to phylum level before analysis. adonis2 function from the vegan R package was used to conduct PERMANOVA.

### Reporting summary

Further information on research design is available in the Nature Research Reporting Summary linked to this article.

### Supplementary information

Supplemental Figures 1-5

Supporting Table S1

Supporting Table S2

Supporting Table S3

Supporting Table S4

Supporting Table S5

Supporting Table S6

Reporting Summary

## Data Availability

The sequencing data are available in NCBI Sequence Read Archive (SRA) repository under BioProject ID PRJNA673773 (SRA accession numbers SAMN16622958 to SAMN16623057) and PRJNA853107 (SRR19856536 to SRR19856993) for water and fish gut data, respectively.
