# Supplementary material for: The intestinal digesta microbiota of tropical marine fish is largely uncultured and distinct from surrounding water microbiota
Source: NPJ Biofilms Microbiomes. 2024 Feb 19;10:11. doi: 10.1038/s41522-024-00484-x (PMC10876542; doi:10.1038/s41522-024-00484-x)
Supplement: Supplementary file 1 — Supplemental Figures 1-5 [file 41522_2024_484_MOESM1_ESM.pdf]

Supplementary Figures

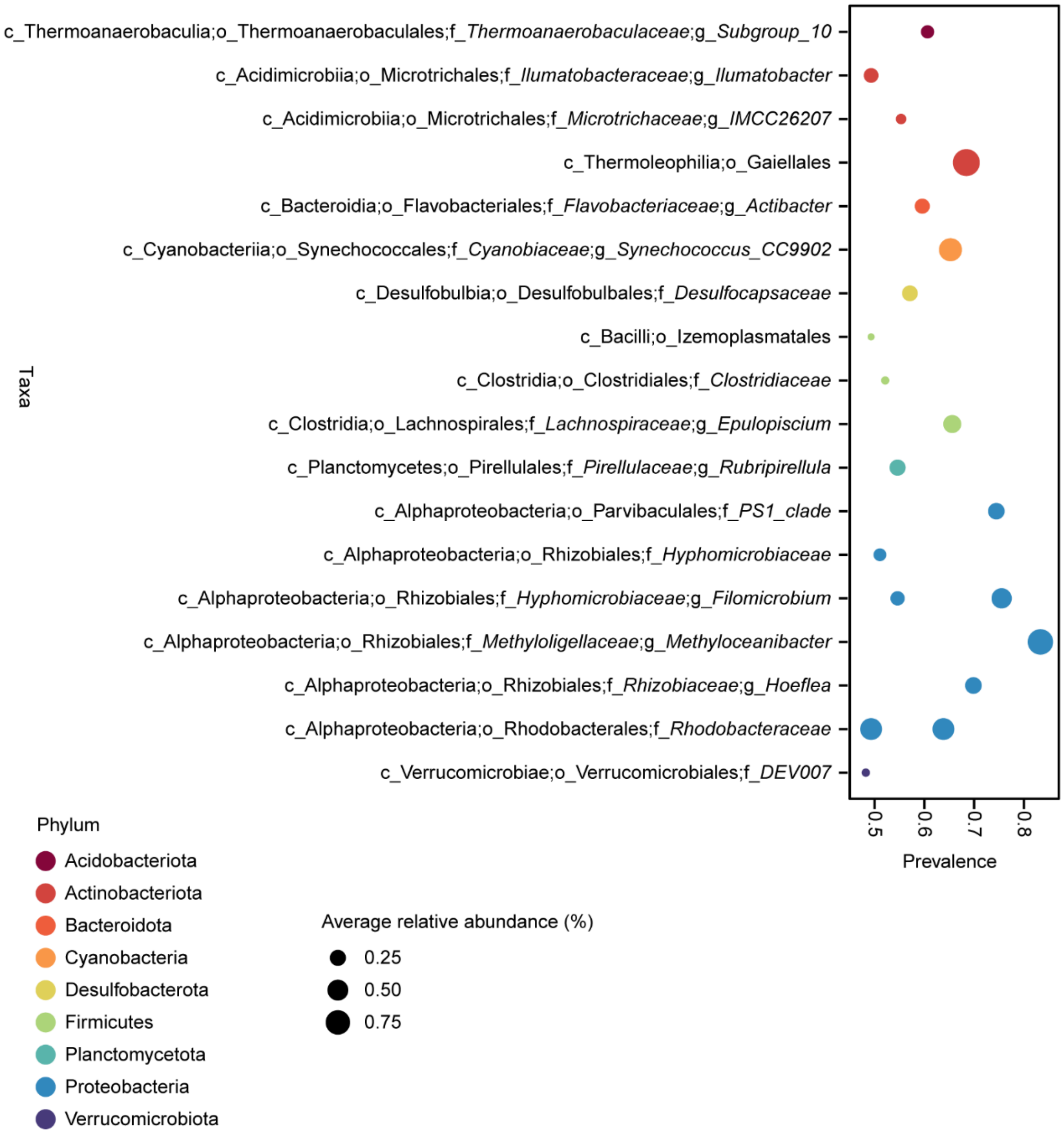

Supplementary Figure 1: Distribution of low abundance microbes in wild fish guts. Each point is one of top 20 most prevalent ASVs that are each less than 1% relative abundance in wild fish gut samples. ASVs ordered based on their closest microbial identification and coloured based on phyla.

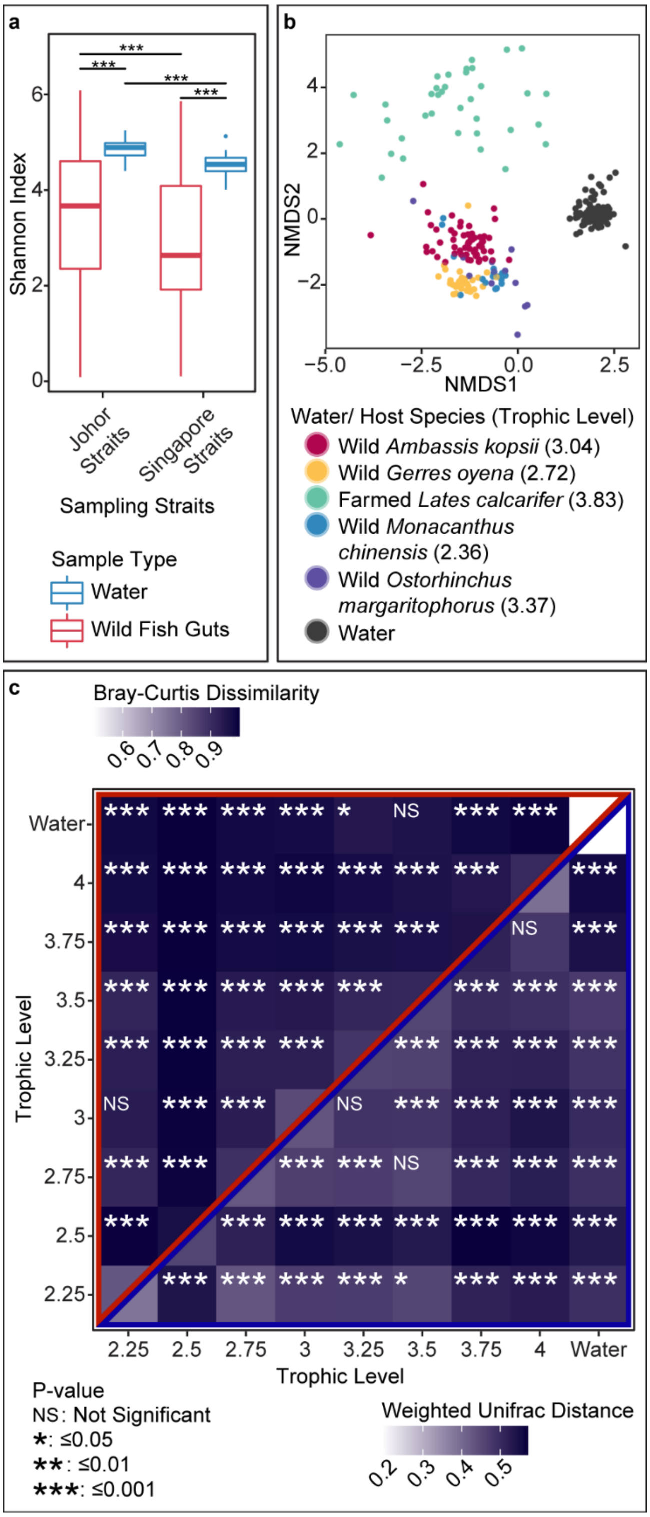

Supplementary Figure 2: Differences in microbial diversity between gut and water samples. A) Boxplots indicating Shannon index of water and gut samples, grouped by sampling location. Box-plot elements are defined are defined as follows: center line, median; box limits, upper

and lower quartiles; whiskers, 1.5x interquartile range; points, outliers. B) Samples were grouped according to host species and domestication status. The five largest groups, where host were identified to species level, and water samples were analysed with NMDS using Bray-Curtis distance. C) Heatmap showing difference in β-diversity between water and wild fish gut samples, where gut samples were grouped by host trophic level, rounded to nearest 0.25. Red triangle represents Bray- Curtis dissimilarity while blue triangle represents weighted Unifrac distance. Wilcoxon rank sum tests were performed within each distance matrix. Benjamini- Hochberg adjusted P-values obtained are indicated on the plot.

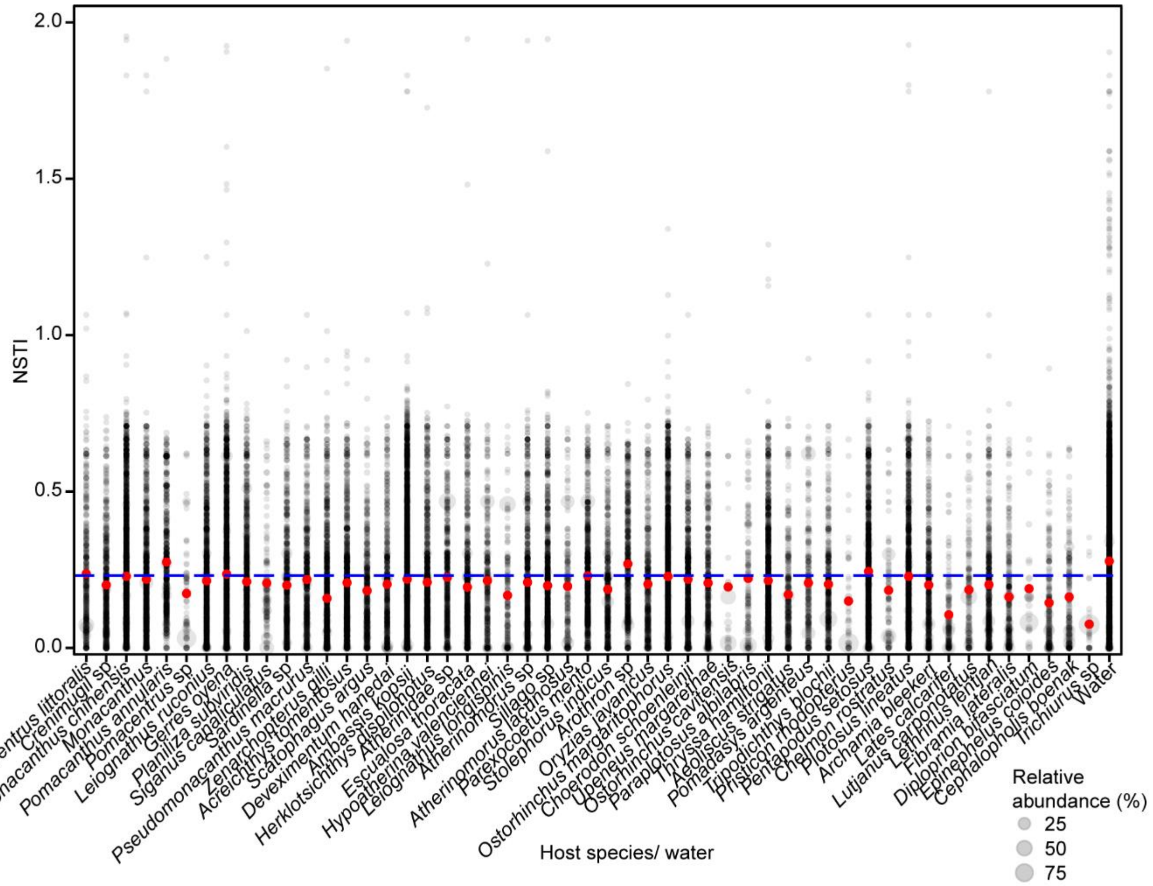

Supplementary Figure 3: ASV novelty compared to isolated genomes across host species. X-axis ordered by host trophic level, from lowest to highest, then water samples. NSTI score for each ASV are indicated by black points where the size of points is determined by relative abundance of ASV in each host species. Red points are mean NSTI for all ASVs in each host species. Horizontal blue dashed line is mean NSTI for all ASVs (mean NSTI = 0.231877).

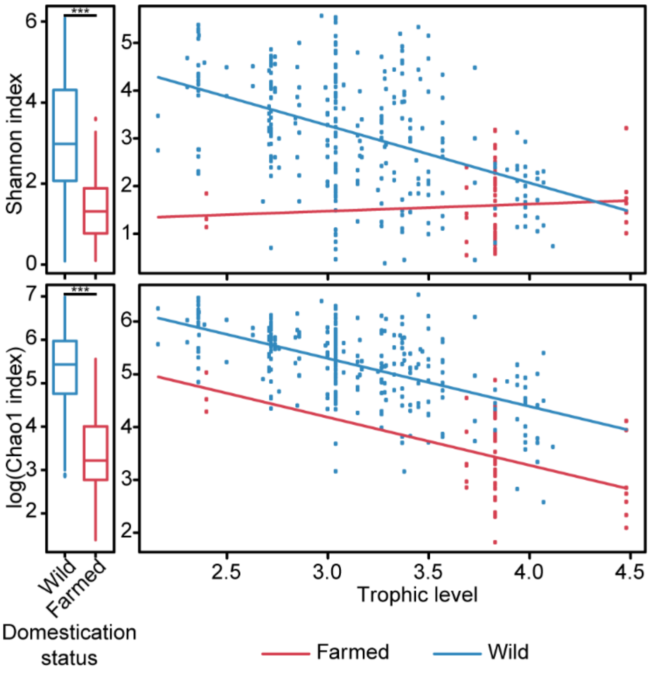

Supplementary Figure 4: α-diversity indices of wild and farmed fish. Boxplots indicate Shannon index and Chao1 index calculated for fish grouped by domestication status. Box-plot elements are defined are defined as follows: center line, median; box limits, upper and lower quartiles; whiskers, 1.5x interquartile range; points, outliers. Boxplots were followed by scatter plots comparing host trophic level against the same α-diversity indices. Points and lines represent samples and linear mixed effect (LME) models respectively. LMEs fit by REML are as follows: Shannon Index~ Trophic Level* Domestication Status+ (1| host species) + (1| Sampling Location) and log(Chao1 Index)~ Trophic Level+ Domestication Status+ (1| host

species) + (1| Sampling Location). AIC values for Shannon index model is 1073.134, and that of Chao1 index model is 757.8932.

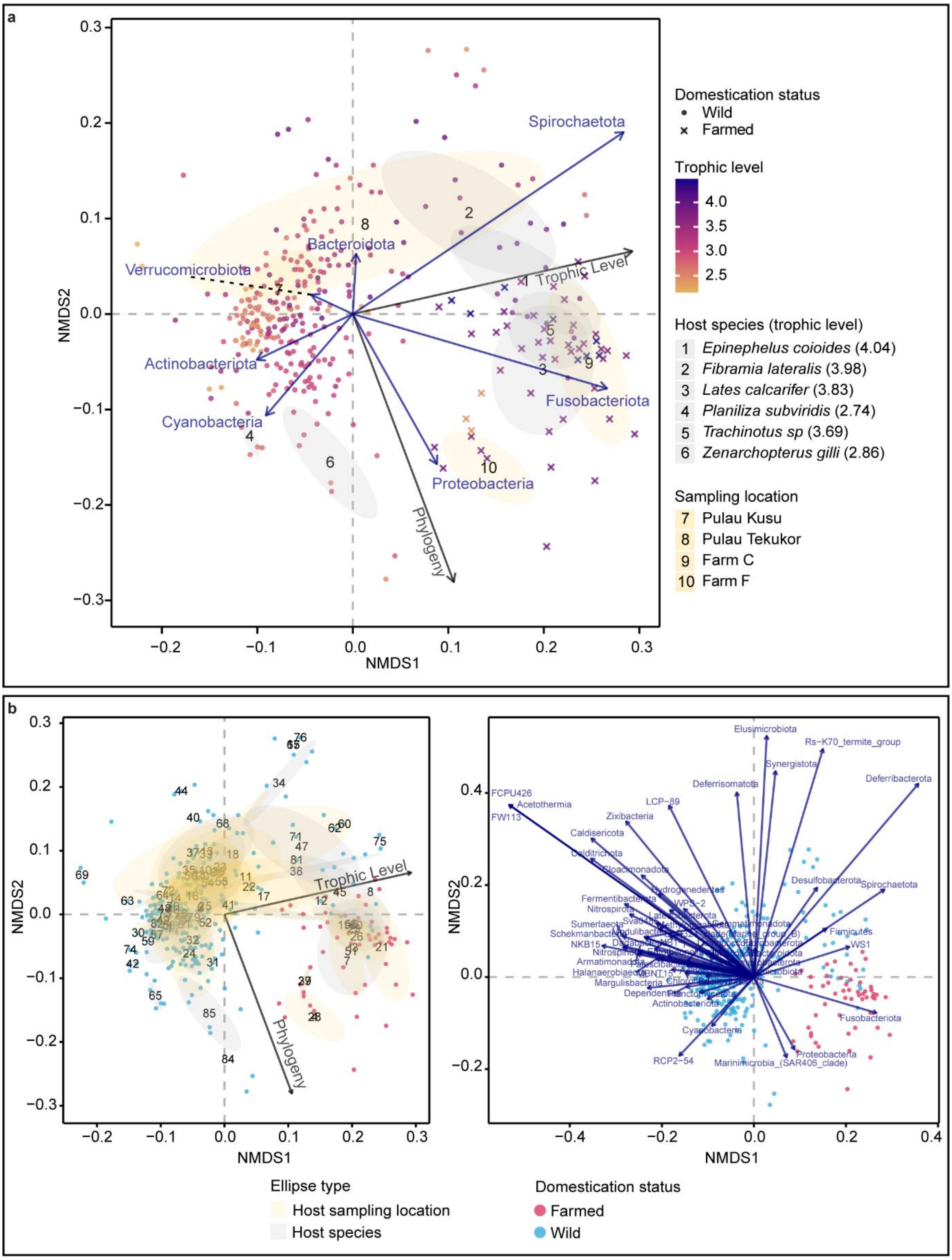

Supplementary Figure 5: Detailed visualization of sample clustering and microbial gradients. NMDS calculated based on Bray- Curtis dissimilarity. Each number represents one centroid, and ellipses indicate standard deviation from centroid. Each grey ellipse represents one host

species, and each yellow ellipse represents one sampling location. Grey arrows indicate effect of two continuous variables. Blue arrows indicate effect of bacterial phyla found in the samples. A) Centroids and vectors were selected as described in Figure 7. B) Left plot illustrates all host species and location ellipses, while right plot contains all phyla-level microbial vectors.

Supplementary Tables

Supplementary table 1: Metadata of fish samples collected. Table includes site descriptions, host measurements, host taxonomy, and database derived host parameters.

Supplementary table 2: Metadata of water samples collected. Table includes site location and habitat type.

Supplementary table 3: Details regarding cultured and uncultured taxa. Taxonomic assignment of ASVs observed in wild, farmed, and water samples and whether there are cultured representatives for each ASV at each taxonomic level.

Supplementary table 4: Power analysis of statistical tests. Total sample sizes are as follows: wild and water samples n=382, wild and water samples without juvenile host n=380, wild and farmed samples n=335. Relevant sample size ranges are presented in the table.

Supplementary table 5: Total number of ASVs that contribute to each pathway shown on the heatmap.

Supplementary table 6: Taxonomic information of each ASV and the pathway each ASV contributes to.
