# Supplementary material for: The intestinal digesta microbiota of tropical marine fish is largely uncultured and distinct from surrounding water microbiota
Source: NPJ Biofilms Microbiomes. 2024 Feb 19;10:11. doi: 10.1038/s41522-024-00484-x (PMC10876542; doi:10.1038/s41522-024-00484-x)
Supplement: Supplementary file 8 — Reporting Summary [file 41522_2024_484_MOESM8_ESM.pdf]

nature portfolio

Corresponding author(s): Henning Seedorf

Last updated by author(s): Feb 2, 2024

Reporting Summary

Nature Portfolio wishes to improve the reproducibility of the work that we publish. This form provides structure for consistency and transparency in reporting. For further information on Nature Portfolio policies, see our Editorial Policies and the Editorial Policy Checklist.

Statistics

For all statistical analyses, confirm that the following items are present in the figure legend, table legend, main text, or Methods section.

n/a	Confirmed	
☐	☒	The exact sample size (*n*) for each experimental group/condition, given as a discrete number and unit of measurement
☐	☒	A statement on whether measurements were taken from distinct samples or whether the same sample was measured repeatedly
☐	☒	The statistical test(s) used AND whether they are one- or two-sided<br>*Only common tests should be described solely by name; describe more complex techniques in the Methods section.*
☐	☒	A description of all covariates tested
☐	☒	A description of any assumptions or corrections, such as tests of normality and adjustment for multiple comparisons
☐	☒	A full description of the statistical parameters including central tendency (e.g. means) or other basic estimates (e.g. regression coefficient) AND variation (e.g. standard deviation) or associated estimates of uncertainty (e.g. confidence intervals)
☐	☒	For null hypothesis testing, the test statistic (e.g. *F*, *t*, *r*) with confidence intervals, effect sizes, degrees of freedom and *P* value noted<br>*Give P values as exact values whenever suitable.*
☒	☐	For Bayesian analysis, information on the choice of priors and Markov chain Monte Carlo settings
☒	☐	For hierarchical and complex designs, identification of the appropriate level for tests and full reporting of outcomes
☐	☒	Estimates of effect sizes (e.g. Cohen's *d*, Pearson's *r*), indicating how they were calculated

Our web collection on statistics for biologists contains articles on many of the points above.

Software and code

Policy information about availability of computer code

Data collection	No software was used for data collection.
Data analysis	QIIME 2 (2021.4), base R, R packages phyloseq (1.44.0) and microbiome (1.22.0), MEGA X (version 10.2.4)

For manuscripts utilizing custom algorithms or software that are central to the research but not yet described in published literature, software must be made available to editors and reviewers. We strongly encourage code deposition in a community repository (e.g. GitHub). See the Nature Portfolio guidelines for submitting code & software for further information.

Data

Policy information about availability of data

All manuscripts must include a data availability statement. This statement should provide the following information, where applicable:

- Accession codes, unique identifiers, or web links for publicly available datasets
- A description of any restrictions on data availability
- For clinical datasets or third party data, please ensure that the statement adheres to our policy

The sequencing data are available in NCBI Sequence Read Archive (SRA) repository under BioProject ID PRJNA673773 (SRA accession numbers SAMN16622958 to SAMN16623057) and PRJNA853107 (SRR19856536 to SRR19856993) for water and fish gut data, respectively.

Research involving human participants, their data, or biological material

Policy information about studies with human participants or human data. See also policy information about sex, gender (identity/presentation), and sexual orientation and race, ethnicity and racism.

Reporting on sex and gender	*Use the terms sex (biological attribute) and gender (shaped by social and cultural circumstances) carefully in order to avoid confusing both terms. Indicate if findings apply to only one sex or gender; describe whether sex and gender were considered in study design; whether sex and/or gender was determined based on self-reporting or assigned and methods used. Provide in the source data disaggregated sex and gender data, where this information has been collected, and if consent has been obtained for sharing of individual-level data; provide overall numbers in this Reporting Summary. Please state if this information has not been collected. Report sex- and gender-based analyses where performed, justify reasons for lack of sex- and gender-based analysis.*
Reporting on race, ethnicity, or other socially relevant groupings	*Please specify the socially constructed or socially relevant categorization variable(s) used in your manuscript and explain why they were used. Please note that such variables should not be used as proxies for other socially constructed/relevant variables (for example, race or ethnicity should not be used as a proxy for socioeconomic status). Provide clear definitions of the relevant terms used, how they were provided (by the participants/respondents, the researchers, or third parties), and the method(s) used to classify people into the different categories (e.g. self-report, census or administrative data, social media data, etc.) Please provide details about how you controlled for confounding variables in your analyses.*
Population characteristics	*Describe the covariate-relevant population characteristics of the human research participants (e.g. age, genotypic information, past and current diagnosis and treatment categories). If you filled out the behavioural & social sciences study design questions and have nothing to add here, write "See above."*
Recruitment	*Describe how participants were recruited. Outline any potential self-selection bias or other biases that may be present and how these are likely to impact results.*
Ethics oversight	*Identify the organization(s) that approved the study protocol.*

Note that full information on the approval of the study protocol must also be provided in the manuscript.

Field-specific reporting

Please select the one below that is the best fit for your research. If you are not sure, read the appropriate sections before making your selection.

☐ Life sciences ☐ Behavioural & social sciences ☒ Ecological, evolutionary & environmental sciences

For a reference copy of the document with all sections, see nature.com/documents/nr-reporting-summary-flat.pdf

Ecological, evolutionary & environmental sciences study design

All studies must disclose on these points even when the disclosure is negative.

Study description	282 wild fish, 53 farm fish, 100 water samples were used. Each sample was amplified in triplicates.
Research sample	Fish and water samples collected around Singapore.
Sampling strategy	For wild samples, all samples were processed. Statistical analysis used could handle sample size differences.
Data collection	Water collection information were recorded by Tay Ywee Chieh. Fish sample information were recorded by Melissa Soh.
Timing and spatial scale	Water sampling was conducted during March and June 2019, timed to coincide with Singapore inter-monsoon and monsoon seasons respectively. Fish were collected periodically between mid 2019 to mid 2021, with a six month gap beginning February 2020 due to pandemic restrictions.
Data exclusions	Samples that did not have sufficient sequencing reads were excluded.
Reproducibility	Each sample was amplified in triplicates, and each single index primer pair was tested with a template-free negative control. Mock microbial community ABRF-MGRG 6 Strain Even Mix Genomic Material (ATCC MSA-3000) was amplified using the same protocol.
Randomization	Randomisation into groups was not applicable in this study. There are no treatment groups/ control groups.
Blinding	Sequencing results were processed together to prevent bias.

Did the study involve field work? ☒ Yes ☐ No

Field work, collection and transport

Field conditions	Samples were collected throughout the year in a tropical climate.
Location	Samples were collected in marine waters around Singapore.
Access & import/export	Boats were rented for off-shore sampling. The full proposal was considered by the IACUC ethics committee, which approved the Biodiversity Protocol under protocol number B17-1123. Wild fishes and water were collected under Permit No:NP/RP18-051b.
Disturbance	Where possible, non-fish organisms were released back to their habitat.

Reporting for specific materials, systems and methods

We require information from authors about some types of materials, experimental systems and methods used in many studies. Here, indicate whether each material, system or method listed is relevant to your study. If you are not sure if a list item applies to your research, read the appropriate section before selecting a response.

Materials & experimental systems

n/a	Involved in the study	
☒	☐	Antibodies
☒	☐	Eukaryotic cell lines
☒	☐	Palaeontology and archaeology
☐	☒	Animals and other organisms
☒	☐	Clinical data
☒	☐	Dual use research of concern
☒	☐	Plants

Methods

n/a	Involved in the study	
☒	☐	ChIP-seq
☒	☐	Flow cytometry
☒	☐	MRI-based neuroimaging

Animals and other research organisms

Policy information about studies involving animals; ARRIVE guidelines recommended for reporting animal research, and Sex and Gender in Research

Laboratory animals	Study did not involve laboratory animals
Wild animals	54 different fish species were captured using seine netting, angling, spear-fishing, or harvested from farms. Fishes were sacrificed, by burial in ice, in order to obtain entire digestive tract.
Reporting on sex	Information was not collected. It is difficult to accurately determine sex of some fish species.
Field-collected samples	Samples were kept in -80oC freezer until DNA extraction.
Ethics oversight	Institutional Animal Care and Use Committee of the National University of Singapore.

Note that full information on the approval of the study protocol must also be provided in the manuscript.
